# Reversing diabetic wound stagnation: macrophage polarization dysregulation as a therapeutic linchpin from pathogenesis to precision interventions

**DOI:** 10.1093/burnst/tkag044

**Published:** 2026-07-12

**Authors:** Shuwen Liu, Chenghao Cai, Huan Liu, Ziyue Zhao, Chunmao Han, Xingang Wang, Yiran Wang

**Affiliations:** Department of Burns and Wound Care Center, the Second Affiliated Hospital of Medical College, Zhejiang University, No. 88 Jiefang Road, Shangcheng District, Hangzhou 310009, China; Zhejiang Key Laboratory of Trauma, Burn, and Medical Rescue, Zhejiang university, No. 866 Yuhangtang Road, Xihu District, Hangzhou 310000, China; Department of Burns and Wound Care Center, the Second Affiliated Hospital of Medical College, Zhejiang University, No. 88 Jiefang Road, Shangcheng District, Hangzhou 310009, China; Zhejiang Key Laboratory of Trauma, Burn, and Medical Rescue, Zhejiang university, No. 866 Yuhangtang Road, Xihu District, Hangzhou 310000, China; Department of Burns and Wound Care Center, the Second Affiliated Hospital of Medical College, Zhejiang University, No. 88 Jiefang Road, Shangcheng District, Hangzhou 310009, China; Zhejiang Key Laboratory of Trauma, Burn, and Medical Rescue, Zhejiang university, No. 866 Yuhangtang Road, Xihu District, Hangzhou 310000, China; Department of Burns and Wound Care Center, the Second Affiliated Hospital of Medical College, Zhejiang University, No. 88 Jiefang Road, Shangcheng District, Hangzhou 310009, China; Zhejiang Key Laboratory of Trauma, Burn, and Medical Rescue, Zhejiang university, No. 866 Yuhangtang Road, Xihu District, Hangzhou 310000, China; Department of Burns and Wound Care Center, the Second Affiliated Hospital of Medical College, Zhejiang University, No. 88 Jiefang Road, Shangcheng District, Hangzhou 310009, China; Zhejiang Key Laboratory of Trauma, Burn, and Medical Rescue, Zhejiang university, No. 866 Yuhangtang Road, Xihu District, Hangzhou 310000, China; Department of Burns and Wound Care Center, the Second Affiliated Hospital of Medical College, Zhejiang University, No. 88 Jiefang Road, Shangcheng District, Hangzhou 310009, China; Zhejiang Key Laboratory of Trauma, Burn, and Medical Rescue, Zhejiang university, No. 866 Yuhangtang Road, Xihu District, Hangzhou 310000, China; Department of Burns and Wound Care Center, the Second Affiliated Hospital of Medical College, Zhejiang University, No. 88 Jiefang Road, Shangcheng District, Hangzhou 310009, China; Zhejiang Key Laboratory of Trauma, Burn, and Medical Rescue, Zhejiang university, No. 866 Yuhangtang Road, Xihu District, Hangzhou 310000, China

**Keywords:** Macrophage polarization, Diabetic wound healing, Chronic inflammation, Immunomodulation, Signalling pathway

## Abstract

The stagnation of diabetic wound healing is a formidable global health challenge that is fundamentally driven by the dysregulation of macrophage plasticity. In the diabetic microenvironment, macrophages remain in a persistent pro-inflammatory (M1) state and fail to transition to the reparative (M2) phenotype essential for tissue regeneration. This review systematically elucidates the molecular pathology of this ‘conversion failure’ from a superficial to an in-depth level and provides a detailed explanation of the mechanisms underlying aberrant macrophage polarization in diabetic wounds, ranging from microenvironmental abnormalities to dysregulated signalling pathways. These perturbations create a vicious cycle of chronic inflammation, impaired angiogenesis, and pathological fibrosis. To address these challenges, we comprehensively outline current therapeutic strategies, including approaches that range from the precision molecular reprogramming of intracellular signalling hubs and gene networks to the engineering of microenvironment-responsive biomaterials capable of neutralizing oxidative stress and responding to pathological cues. Furthermore, we highlight the integration of exogenous bioactivity through stem cell– and exosome-based therapies aimed at replenishing the regenerative niche. Additionally, we critically assess translational bottlenecks, suggesting a paradigm shift from the binary M1/M2 model towards targeting intermediate phenotypes identified by single-cell multiomics. By integrating mechanistic insights with advanced immunomodulatory engineering, this review provides a theoretical framework for developing next-generation precision therapies to reverse the chronic nature of diabetic wounds.

## Highlights

- Elucidates the ‘metabolic–epigenetic locking’ and ‘conversion failure’ mechanisms that restrain macrophages in a persistent M1 state within the diabetic wound microenvironment.
- Systematically analyses how impaired hypoxic signalling, oxidative stress, and neuropathy-induced calcitonin gene-related peptide deficiency disrupt the activity of intracellular signalling hubs (NF-κB, HIF, and JAK–STAT) to block M2 polarization.
- Classifies emerging strategies into precision molecular reprogramming, microenvironment normalization (smart responsive systems), and exogenous bioactivity supplementation to address suppressed signalling and microenvironmental chaos.
- Advocates for a paradigm shift from the binary M1/M2 model towards targeting intermediate ‘transcriptionally stalled’ phenotypes identified by single-cell multiomics for next-generation precision therapies.

## Background

Diabetes is a significant global health challenge, the prevalence of which has been steadily increasing since the 1990s. By 2021, the number of individuals affected by diabetes reached 529 million worldwide, and this figure is projected to increase to 1.31 billion by 2050. The high prevalence of diabetes, coupled with its associated complications, imposes a substantial burden on health care systems and economies worldwide [1, 2]. Owing to the combined effects of chronic hyperglycaemia, microcirculation disorders, impaired immune function, neuropathy, and infections, diabetic patients are particularly prone to chronic traumatic or ulcerative wounds that are difficult to heal or heal slowly. Notably, diabetic foot ulcers (DFUs) are recognized as a common and severe complication [3–5]. It is estimated that the lifetime risk of foot ulcers in diabetic patients ranges from 19% to 34%, with recurrence rates as high as 65% within 3 to 5 years, accompanied by increased rates of amputation and mortality [6, 7]. Despite advancements in modern medicine in terms of glycaemic control and wound care, ~30% of DFU patients exhibit treatment resistance [8], suggesting inherent limitations in conventional therapeutic strategies that focus primarily on microbial control and vascular reconstruction.

Chronic diabetic wounds involve multiple complex pathophysiological processes, and recent studies have identified immune microenvironment dysregulation as a central mechanism underlying impaired wound healing in diabetes patients. As key regulators of the innate immune system, macrophages exhibit distinct spatiotemporal regulatory properties during wound healing. Under normal physiological conditions, a dynamic balance between M1 (pro-inflammatory) and M2 (anti-inflammatory and pro-repair) macrophages is maintained. M1 macrophages play critical roles in the early phase of wound healing by promoting inflammatory responses and clearing pathogens, wound debris, and apoptotic cells. In the later repair phase, macrophages transition to the M2 phenotype to suppress inflammation and release cytokines and growth factors that modulate the proliferation, differentiation, and migration of keratinocytes, fibroblasts, and endothelial cells, thereby promoting angiogenesis and wound closure [9, 10]. However, the complex microenvironment of diabetic wounds disrupts the macrophage polarization program. Factors such as hyperglycaemia, hypoxia, and infection lead to macrophages exhibiting an M1 phenotype for a prolonged duration through various mechanisms. This imbalance in macrophage polarization sustains chronic inflammation, resulting in excessive secretion of pro-inflammatory cytokines (e.g. TNF-α and IL-1β) and insufficient production of anti-inflammatory cytokines (e.g. IL-4 and IL-13) and reparative growth factors (e.g. TGF-β1 and VEGF), ultimately forming a vicious cycle that hinders wound repair [9, 11, 12].

**Figure 1:**
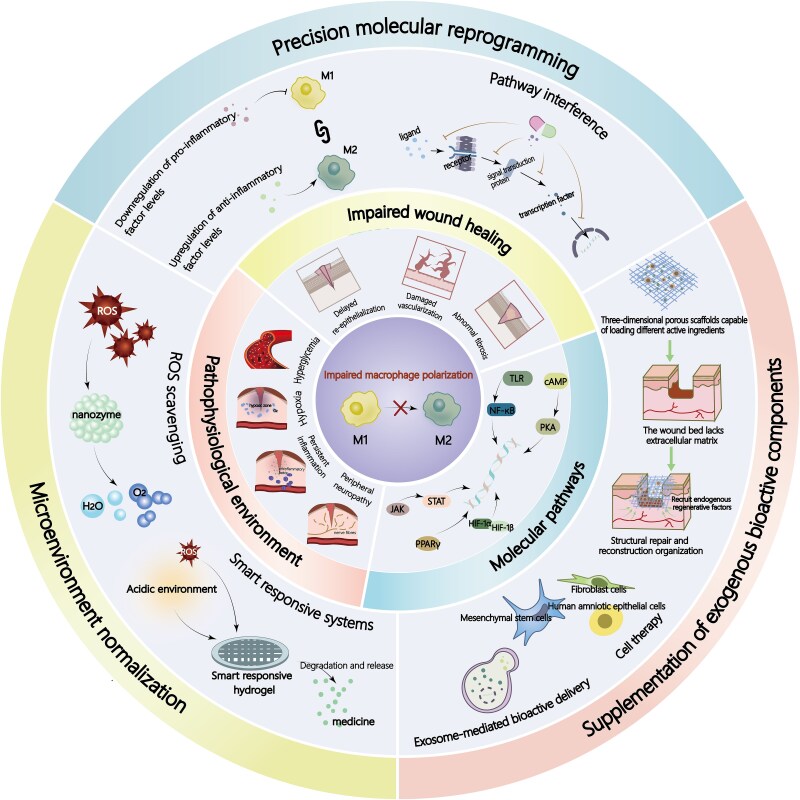
Dysregulated macrophage polarization represents a central mechanism underlying impaired diabetic wound healing. The dysregulated diabetic wound microenvironment drives aberrant macrophage polarization, thereby triggering a cascade of pathophysiological alterations and healing dysfunction, with multiple signalling pathways mechanistically implicated. Therapeutic strategies targeting macrophage polarization dysregulation have demonstrated critical efficacy in managing diabetic wounds. *M1* M1 macrophage, *M2* M2 macrophage, *ROS* reactive oxygen species, *TLR* toll-like receptor, *NF-κB* nuclear factor kappa B, *cAMP* cyclic adenosine monophosphate, *PKA* protein kinase A, *JAK* Janus kinase, *STAT* signal transducer and activator of transcription, *PPARγ* peroxisome proliferator-activated receptor gamma, *HIF-1α* hypoxia-inducible factor 1-alpha, *HIF-1β* hypoxia-inducible factor 1-beta

Given the critical role of macrophage polarization imbalance in the impaired healing of diabetic wounds, targeting macrophage phenotypic reprogramming has emerged as a cutting-edge focus of diabetic wound therapy research [13]. This review provides a comprehensive analysis of the dual regulatory role of macrophage polarization in diabetic wound healing, with a particular emphasis on the dysfunction of macrophages in diabetic wounds, the molecular mechanisms underlying chronic wound formation, and therapeutic interventions aimed at restoring macrophage function (Figure 1). The objective is to lay a theoretical foundation for overcoming the challenges associated with treating chronic diabetic wounds.

## Review

### Biological basis of macrophage polarization

Macrophages are indispensable components of the human immune system. In most adult tissues, they originate from two distinct pathways: one subset is derived from embryonic progenitor cells, such as primitive haematopoietic stem cells in the yolk sac, while another subset develops from bone marrow–derived blood monocytes. These monocytes migrate through the bloodstream to tissues, where they settle and subsequently differentiate into mature macrophages [14–16]. Macrophages perform diverse functions, including phagocytosis and pathogen clearance, antigen presentation, and immune regulation [17–20], and participate in numerous biological processes, such as modulating endogenous reactive oxygen species (ROS) levels, maintaining iron homeostasis, facilitating tissue damage repair, and regulating metabolic functions [21]. Macrophages, primarily tissue-resident macrophages (TRMs) and wound-associated macrophages (WAMs), play critical roles throughout all phases of wound healing. Following skin injury, blood monocytes are recruited to the wound site under inflammatory or stress conditions, where microenvironmental signals induce their differentiation into macrophages. Furthermore, TRMs originating from yolk sac–derived embryonic precursors are early responders that maintain skin homeostasis [15, 17, 22–24]. Moreover, macrophages serve as pivotal coordinators during tissue repair by regulating the behaviour of multiple cell lineages, including fibroblasts, vascular endothelial cells, and less-studied populations such as adipocytes and melanocytes, ultimately promoting wound closure and tissue remodelling [25].

Macrophages exhibit high plasticity and can alter their phenotype in response to diverse environmental stimuli [23, 26]. Macrophage polarization refers to the process by which macropha-ges adopt distinct functional states and phenotypes under specific microenvironmental signals. This differentiation enables macrophages to rapidly adapt to external conditions, thereby fulfilling diverse physiological and pathological roles [10, 26]. The two primary macrophage subsets are classically activated (pro-inflammatory, M1) and alternatively activated (anti-inflammatory, M2) macrophages (Table 1). M1 macrophages, also termed classically activated macrophages, are typically induced by IFN-γ and TNF-α secreted by CD4^+^ T helper 1 (Th1) cells or by the recognition of lipopolysaccharides (LPSs) from gram-negative bacteria. These macrophages produce and secrete high levels of pro-inflammatory cytokines, including TNF-α, IL-1α, IL-1β, IL-6, IL-12, IL-23, and cyclooxygenase-2 (COX-2), along with nitric oxide (NO) and chemokine ligands such as CXCL9 and CXCL10 [23, 27]. The release of these pro-inflammatory mediators can induce ROS-mediated tissue damage, thereby impairing wound healing [28]. M1 macrophages play critical roles in immune activation, inflammation promotion, antibacterial activity, anti-tumour immunity, and host defence [21]. M2 macrophages, or alternatively activated macrophages, suppress inflammation. They are polarized via activation of the JAK–STAT pathway by Th2 cytokines (e.g. IL-4 and IL-13) or other anti- inflammatory cytokines (e.g. IL-10). M2 macrophages produce anti-inflammatory factors such as IL-10 and TGF-β [23, 27], and their functions include decreasing the levels of inflammatory cytokines and ROS, increasing the levels of anti-inflammatory cytokines (e.g. TGF-β, IL-10, IL-1 receptor antagonist, and IL-1 type II decoy receptor), and secreting growth factors such as platelet-derived growth factor (PDGF), epidermal growth factor, and vascular endothelial growth factor (VEGF) [17]. These cells are pivotal for wound healing, parasitic responses, hypersensitivity, angiogenesis, tissue remodelling, and immune suppression [21]. On the basis of their inducing factors and functional characteristics, M2 macrophages can be further categorized into different subtypes (Table 2). This classification provides a critical framework for investigating the mechanistic roles of M2 macrophages in pathological contexts and identifying therapeutic targets [27, 29–31].

**Table 1:** Summary of differences between M1 and M2 macrophages

Feature	M1 macrophages	M2 macrophages
Activation	Pro-inflammatory signals: LPS, IFN-γ	Anti-inflammatory or immunoregulatory signals: IL-4, IL-13, IL-10, glucocorticoids
Secreted cytokines	TNF-α, IL-1β, IL-6, IL-12, IL-23, ROS, NO	IL-10, TGF-β, CCL17, CCL22, Arg-1, VEGF
Surface markers	CD80, CD86, MHC-II, iNOS	CD206 (MR), CD163, CD204 (SR-A), Arg-1, CD301 (MGL), Stabilin-1, TREM2
Primary functions	Pro-inflammatory responses, pathogen killing, anti-tumour immunity, tissue damage	Anti-inflammatory responses, tissue repair, immune regulation, angiogenesis, tumour microenvironment support (pro-tumorigenic)
Metabolic profile	Enhanced glycolysis; produces ROS and NO	Enhanced oxidative phosphorylation and fatty acid oxidation; arginine metabolism
Key transcription factors	STAT1, NF-κB, IRF5	STAT6, PPARγ, IRF4
Typical context	Early acute infections, Th1 immune responses	Chronic inflammation resolution, parasitic infections, allergy, wound healing

*Arg-1* arginase-1, *CCL17* C-C motif chemokine ligand 17, *CCL22* C-C motif chemokine ligand 22, *IFN-γ* interferon-gamma, *IL* interleukin, *iNOS* inducible nitric oxide synthase, *IRF4/5* interferon regulatory factor 4/5, *LPS* lipopolysaccharide, *MGL* macrophage galactose-type lectin, *MHC-II* major histocompatibility complex class II, *MR* mannose receptor, *NF-κB* nuclear factor kappa B, *NO* nitric oxide, *PPARγ* peroxisome proliferator-activated receptor gamma, *ROS* reactive oxygen species, scavenger receptor A, *STAT1/6* signal transducer and activator of transcription 1/6, *TGF-β* transforming growth factor-beta, *Th1* T helper cell type 1, *TNF-α* tumour necrosis factor-alpha, *TREM2* triggering receptor expressed on myeloid cells 2, *VEGF* vascular endothelial growth factor

**Table 2:** Classification and functional profiling of M2 macrophage subtypes

Subtype	Primary inducing factors	Key markers/molecules	Primary functional characteristics
M2a	IL-4, IL-13 (*via* IL-4Rα/Stat6 pathway)	CD206 (mannose receptor), Arg-1, Ym1/2 (rodents), Fizz1 (RELMα), CCL17, CCL18, CCL22	Anti-inflammatory, promotes tissue repair and fibrosis, enhances wound healing, stimulates ECM deposition, anti-parasitic immunity, suppresses Th1 responses
M2b	Immune complexes + TLR agonists (e.g. LPS) or IL-1β	High IL-10, low IL-12, IL-1β, TNFα, CCL1	Immunoregulatory, promotes Th2 responses, expands regulatory B cells, mixed pro-inflammatory and anti-inflammatory properties (initial pro-inflammation shifts to regulation)
M2c	IL-10, glucocorticoids, TGF-β	CD163 (haemoglobin scavenger receptor), Mer receptor, Arg-1 (partial), TGF-β, CCL16	Promote anti-inflammatory, immunosuppressive, efficient efferocytosis (clearance of apoptotic cells), promotes tissue remodelling, suppresses T-cell responses
M2d	Adenosine, TLR antagonists (e.g. IL-10, TGF-β, PGE₂), hypoxia, tumour microenvironment factors	High Arg-1, IL-10, VEGF, TGF-β, Tie-2 receptor, low/absent IL-12	Promotes angiogenesis (high VEGF), promotes tumour growth, invasion and metastasis, suppresses anti-tumour immunity (T cells), tissue remodelling

*Arg-1* arginase-1, *CCL* C-C motif chemokine ligand. *CD* cluster of differentiation, *ECM* extracellular matrix, *Fizz1* found in inflammatory zone 1, *IC* immune complexes, *IL* interleukin, *LPS* lipopolysaccharide, *PGE₂* prostaglandin E2, *RELMα* resistin-like molecule alpha, *RXR* retinoid X receptor, *TGF-β* transforming growth factor-beta, *TLR* Toll-like receptor, *TNFα* tumour necrosis factor-alpha, *VEGF* vascular endothelial growth factor

### The macrophage response is impaired during diabetic wound healing

#### Abnormal macrophage polarization in diabetic wounds: M1 skewing and M2 suppression

**Figure 2:**
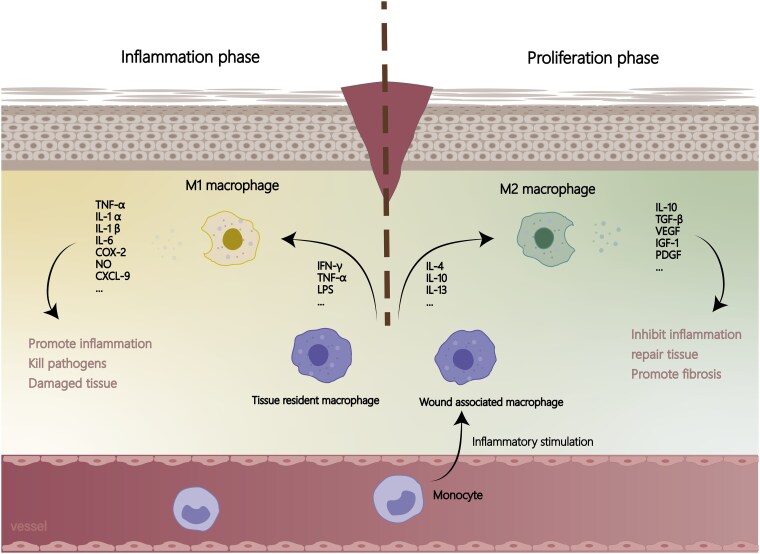
Macrophage polarization during normal wound healing. Macrophages are categorized into tissue-resident macrophages (TRMs) and wound-associated macrophages (WAMs). During normal wound healing, these macrophages undergo dynamic phenotypic transitions: In the early phase (Days 1–3), the M1 phenotype, marked by CD86, iNOS, and TNF-α, mediates pro-inflammatory and antimicrobial effects. By the later phase (around Day 7), macrophages shift to the M2 phenotype, secreting factors such as IL-10 and TGF-β to promote tissue repair, remodelling, and homeostasis. *COX-2* cyclooxygenase-2, *CXCL-9* C-X-C motif chemokine ligand 9, *IFN-γ* interferon-gamma, *IGF-1* insulin-like growth factor 1, *IL* interleukin, *LPS* lipopolysaccharide, *NO* nitric oxide, *PDGF* platelet-derived growth factor, *TGF-β* transforming growth factor-beta, *TNF-α* tumour necrosis factor-alpha, *VEGF* vascular endothelial growth factor

During normal wound healing, macrophages infiltrating the wound site are activated and adopt the M1 phenotype, which is characterized by the expression of specific markers such as CD86, inducible nitric oxide synthase (iNOS), and TNF-α. These M1 macrophages dominate from Days 1 to 3 and release pro-inflammatory factors, including TNF-α, IL-1β, IL-12, and IL-23, which mediate pro-inflammatory and antimicrobial functions. The M1 phenotype subsequently gradually transitions towards the M2 phenotype, which peaks at approximately Day 7. M2 macrophages secrete large amounts of anti-inflammatory factors such as IL-10 and TGF-β, which suppress inflammation, promote tissue repair and remodelling, facilitate angiogenesis, and maintain homeostasis (Figure 2) [23, 28, 32, 33]. This phenotypic transition of macrophages plays a critical role in the regulation of inflammation during wound healing. Any disruption in this process may lead to stagnation of the inflammatory phase, resulting in chronic nonhealing wounds [17, 24].

In the context of diabetes, however, this tightly choreographed temporal sequence is fundamentally disrupted. Unlike repair under physiological conditions in which inflammation is transient and self-resolving, diabetic wounds are characterized by a ‘failure to switch’. The diabetic microenvironment promotes the persistence of pro-inflammatory M1 macrophages, preventing their timely transition to the reparative M2 phenotype essential for tissue reconstruction. This imbalance perpetuates chronic inflammation, impairs tissue regeneration, delays wound healing, and may even result in gangrene and subsequent amputation [34, 35]. The diabetic wound microenvironment is initially characterized by an exaggerated inflammatory response, as evidenced by a significantly elevated CCR7/CD68 ratio in the dermis on Day 3 postinjury compared with that in the normal dermis [33]. Crucially, this pro-inflammatory state fails to resolve, and by Day 7, when a transition to the reparative phenotype is typically expected, diabetic wounds instead exhibit persistent M1 macrophage dominance. Quantitative analyses have confirmed that the levels of M1 macrophages at this stage remain significantly higher than both the intrinsic M2 macrophage population and those detected in nondiabetic individuals [36–38], indicating fundamental arrest of the M1-to-M2 transition. Further isolation and quantitative analysis of myeloid cells in wounds revealed distinct differences in the subtypes and morphological profiles of myeloid cell populations between diabetic and normal wounds, including the aberrant recruitment, retention, and differentiation of inflammatory cells in diabetic wounds during the early inflammatory phase [36]. Biopsy samples from the wound margins of human diabetic foot ulcers demonstrated a significant reduction in the number of M2 macrophages in nonhealing ulcers, with no notable difference in the numbers of M1 macrophages. These findings underscore a definitive link between M2 polarization and healing efficacy, supporting the concept that impaired polarization towards the M2 phenotype is a critical determinant of poor diabetic wound healing outcomes [36]. Collectively, these findings highlight that diabetic wounds exhibit aberrant macrophage polarization or hyperpolarization, wherein early-phase M1 macrophages fail to transition to the M2 phenotype and persist in the wound microenvironment. This pathological state, characterized by M1 skewing and M2 suppression, represents a key mechanism underlying the impaired healing of diabetic wounds. However, the classical M1/M2 dichotomy is being increasingly recognized as an oversimplification, particularly in the complex diabetic wound microenvironment. Recent single-cell RNA sequencing (scRNA-seq) studies have revealed a continuum of macrophage activation states rather than two distinct phenotypes. In diabetic wounds, macrophages often exhibit a ‘mixed’ or ‘discordant’ phenotype (coexpressing M1 markers such as TNF-α and M2 markers such as Arg1), indicating a state of ‘transcriptional stall’ or ‘plasticity arrest’ distinct from canonical polarization. Therefore, modern immunology is increasingly abandoning the term ‘polarization’ and preferring to use ‘activation state’ or ‘functional phenotype’ to describe this heterogeneity [39]. Understanding this nonbinary spectrum is crucial, as therapeutic strategies targeting a simple M1-to-M2 switch may fail to address these intermediate, dysfunctional subpopulations.

#### Impact of the diabetic wound environment on macrophage phenotypic switching

**Figure 3:**
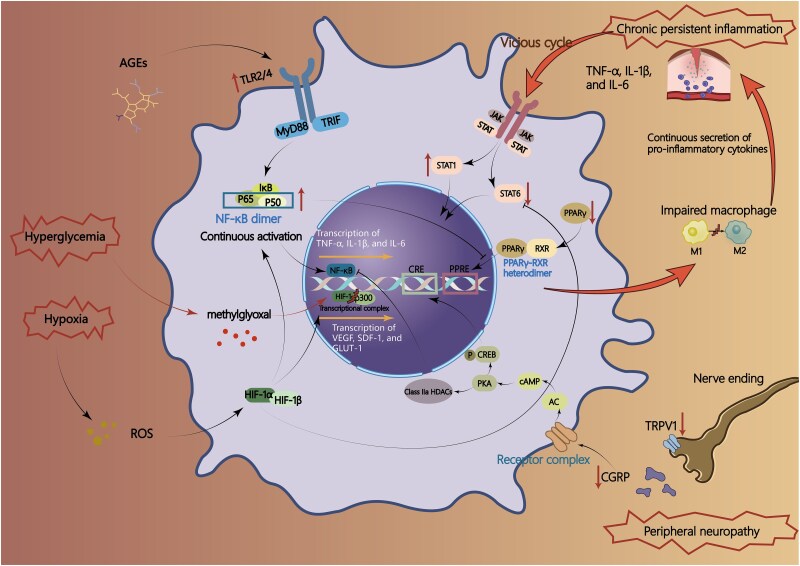
The intricate microenvironment of diabetic wounds and relevant signalling pathways disrupt macrophage polarization balance. The intricate microenvironment of diabetic wounds, characterized by hyperglycaemia, hypoxia, persistent chronic inflammation, and neuropathy, directly disrupts macrophage polarization balance through pathways such as NF-κB, HIF, PPARγ, JAK–STAT, and cAMP-PKA, or indirectly influences it via extensive crosstalk among these pathways. Among these factors, the sustained activation of M1-type macrophages continuously secretes pro-inflammatory cytokines, further exacerbating wound inflammation, thereby creating a vicious cycle of chronic and persistent inflammation in diabetic wounds.*AC* adenylate cyclase, *AGEs* advanced glycation end-products, *cAMP* cyclic adenosine monophosphate, *CGRP* calcitonin gene-related peptide, *CRE* cAMP response element, *p-CREB* phosphor-cAMP response element-binding protein, *GLUT-1* glucose transporter 1, *HDACs* histone deacetylases, *HIF-1α* hypoxia-inducible factor 1-alpha, *HIF-1β* hypoxia-inducible factor 1-beta, *IL* interleukin, *IκB* inhibitor of kappa B, *NF-κB* nuclear factor kappa B, *PKA* protein kinase A, *PPARγ* peroxisome proliferator-activated receptor gamma, *PPRE* peroxisome proliferator-activated receptor response element, *ROS* reactive oxygen species, *RXR* retinoid X receptor, *SDF-1* stromal cell-derived factor-1, *STAT1/6* signal transducer and activator of transcription 1/6, *TLR2/4* toll-like receptor 2/4, *TNF-α* tumour necrosis factor-alpha, *TRIF* TIR-domain-containing adapter-inducing interferon-β, *TRPV1* transient receptor potential vanilloid 1

The microenvironment of diabetic wounds is exceedingly complex, as multiple factors have been proven to have detrimental effects on the M1/M2 phenotype transition. These include metabolism-related outcomes such as hyperglycaemia and the production of advanced glycation end-products (AGEs) and oxidative stress byproducts, as well as soluble molecules such as inflammatory cytokines and neuropeptides [5]. This section provides a detailed discussion of the diabetic wound microenvironment and associated signalling pathways, revealing the underlying causes of aberrant macrophage polarization in diabetic wounds (Figure 3).

##### Hyperglycaemia-driven oxidative stress and NF-κB activation

In diabetic wounds, macrophage dysfunction is not merely a local event but begins systemically. Hyperglycaemia modulates gene expression in haematopoietic stem and progenitor cells within the bone marrow, imprinting an ‘inflammatory memory’ that biases differentiation towards M1-like phenotypes even before monocytes infiltrate the tissue [5, 17, 35, 40]. Upon recruitment to the wound, this predisposition is exacerbated by the hyperglycaemic microenvironment. *In vitro* studies have confirmed that human monocytes (THP-1 cells) cultured under high-glucose conditions upregulate M1 markers (e.g. CCR7) while downregulating the expression of the reparative cytokine TGF-β1 [33, 41]. Microarray analyses revealed the broad upregulation of pro-inflammatory cytokines and chemokines, establishing a vicious cycle in which macrophages perpetuate inflammation rather than resolve it [41, 42].

Functionally, this imbalance in macrophage polarization manifests as a critical defect in phagocytosis, specifically, the failure to clear apoptotic neutrophils (efferocytosis). Diabetic macrophages exhibit marked downregulation of the expression of the scavenger receptor CD36 and αvβ3 integrin [42, 43]. This prevents the clearance of necrotic debris and pathogens, leading to secondary necrosis and persistent inflammatory signalling [44–46]. Notably, this phagocytic defect is sustained throughout the healing process in diabetic mice, unlike the transient decline seen in nondiabetic controls [43].

The molecular drivers of these phenotypic and functional deficits are AGEs and ROS, which act as bridges to intracellular pathway dysregulation. AGEs accumulate in diabetic tissues and bind to their receptor (RAGE), not only triggering pro-inflammatory cytokine release but also hijacking macrophage autophagy. Studies have indicated that AGE-induced autophagy promotes M1 polarization, whereas inhibition of this process restores M2 macrophage homeostasis [47–50]. The AGE–RAGE interaction activates NADPH oxidase, generating excessive amounts of ROS. This oxidative stress forms a positive feedback loop with AGE formation, amplifying tissue damage [48, 51–53]. Furthermore, the hyperglycaemic microenvironment induces time- and dose-dependent upregulation of the expression of TLRs, particularly TLR2 and TLR4 [54, 55]. While TLR expression is typically transient during the inflammatory phase of normally healing wounds, diabetic wounds exhibit persistently elevated levels of these receptors from injury through the repair phases [24]. This sustained overexpression sensitizes macrophages to both pathogen-associated molecular patterns and endogenous damage-associated molecular patterns released by necrotic cells, inducing a second, relentless stream of pro-inflammatory signalling in addition to that originating from the AGE–RAGE pathway.

Critically, these upstream signals, namely, metabolic stressors (AGEs/ROS) and receptor hyperactivation (TLR2/4), converge and lead to the constitutive activation of the NF-κB signalling hub, transforming chemical insults into sustained transcriptional blockade [56]. While NF-κB activation is typically transient during physiological healing processes, the diabetic milieu, which is rich in ROS, AGEs, and TLR ligands, triggers the persistent phosphorylation of IκB and nuclear translocation of the p65 subunit [57, 58]. This p65 subunit is the specific driver of M1 polarization that directly initiates the transcription of TNF-α, IL-1β, and IL-6 while suppressing M2 macrophage-associated genes [58, 59]. Furthermore, this activation is not easily reversible because of ‘metabolic–epigenetic locking’. Hyperglycaemic memory manifests as persistent epigenetic modifications (e.g. H3K4me1) of the NF-κB promoter, maintaining hyperactivity of the pathway even after glycaemic control is restored [60–62]. This mechanism explains why targeting downstream cytokines often fails; the macrophage is genetically ‘locked’ in a pro-inflammatory state, preventing the essential transition to the M2 reparative phenotype.

##### Hypoxic microenvironment and impaired hypoxic signalling

The hypoxic microenvironment is a characteristic pathological feature of diabetic wounds that results from the interplay of multiple factors, including impaired peripheral perfusion, metabolic disturbances, and the high oxygen demand of the inflammatory response [42]. Under physiological conditions, this decrease in oxygen tension stabilizes hypoxia-inducible factor 1 (HIF-1), a master transcription factor composed of the HIF-1α and HIF-1β subunits. Stabilized HIF-1α is translocated to the nucleus and drives the expression of genes essential for survival, such as VEGF (angiogenesis), GLUT-1 (glucose uptake), and those encoding glycolytic enzymes [63, 64]. HIF-1α promotes macrophage polarization towards the M1 phenotype and drives macrophage metabolic reprogramming from oxidative phosphorylation to glycolysis. This metabolic shift is critical for the rapid generation of ATP and ROS by M1 macrophages for pathogen clearance [65–67]. Mechanistically, HIF-1α interacts with factors in the NF-κB pathway, where stabilized HIF-1α can directly increase NF-κB transcriptional activity, thereby amplifying the production of inflammatory cytokines such as TNF-α and IL-6 and promoting M1 polarization [68–71]. Moreover, HIF-1α inhibits the JAK–STAT pathway, which drives macrophage polarization towards the M2 phenotype. HIF-1α also suppresses STAT6 phosphorylation or competes for essential co-activators such as p300, effectively blocking the IL-4-driven transition towards a reparative phenotype [72]. This HIF-mediated cross-regulation that activates NF-κB while suppressing STAT6 creates a rigid barrier that inhibits macrophage reprogramming, locking cells in a chronic inflammatory state.

However, in diabetic wounds, the HIF-1 signalling pathway fails to respond effectively even in the presence of severe tissue hypoxia. This impairment is associated with chronic hyperglycaemia, which leads to the overproduction of methylglyoxal. This metabolic byproduct chemically modifies the transcriptional co-activator p300, disrupting its interaction with HIF-1α. The consequent failure to form a functional transcription complex prevents the activation of downstream target genes, rendering the wounded tissue ‘blind’ to its own hypoxic state and severely compromising angiogenesis and metabolic adaptation [64, 73]. Specifically, cellular energy status is a determinant of macrophage fate; ATP levels are crucial for M2 differentiation because JAK–STAT6 signalling, which is central to M2 polarization, depends on intracellular ATP availability. Under physiological conditions, high HIF-1 activity during the hypoxic pro-inflammatory phase plays a decisive role in driving macrophage metabolic reprogramming towards glycolysis, enabling sufficient ATP production in the oxygen-deprived environment, thereby supporting a shift towards M2 dominance as inflammation resolves or the repair phase begins. In diabetic wounds, however, aberrant HIF signalling disrupts this balance of macrophage polarization [74–76].

Conclusively, this functional uncoupling, where tissue is hypoxic but HIF-1α fails to stabilize, represents a critical point of failure. This suggests that simply adding VEGF (a downstream target) is a short-term solution. The fundamental challenge lies in restoring the oxygen-sensing machinery itself (e.g. preventing the methylglyoxal-mediated modification of p300). Future interventions must distinguish between ‘promoting hypoxic signalling’ and ‘ameliorating tissue hypoxia’, as these outcomes require distinct therapeutic approaches.

##### Chronic inflammatory microenvironments and signalling feedback loops

Chronic low-grade systemic inflammation, which is a hallmark of ageing, obesity, and diabetes, is associated with increased levels of pro-inflammatory macrophages. Prolonged systemic inflammation can be destructive to tissues and can impair wound healing [5, 77].

Diabetic wounds are fundamentally characterized by a sustained inflammatory response and a pathological deviation from the normal healing trajectory. Unlike the transient inflammation seen during physiological wound repair, the diabetic microenvironment is characterized by chronic low-grade systemic inflammation [5, 77]. At the genetic level, RNA sequencing and rigorous bioinformatics analysis revealed four immune-related genes (MMP9, IL1B, ICAM1, and CXCL8) that were significantly upregulated in DFU patients compared with healthy controls and strongly associated with inflammatory processes [78]. This creates a toxic milieu with an imbalanced cytokine profile defined by the excessive release of pro-inflammatory mediators such as TNF-α, IL-1β, and IL-6 and the suppression of anti-inflammatory factors such as IL-10 and TGF-β. This ‘cytokine storm’ is not merely a symptom but a driver that actively disrupts intracellular signalling networks.

In the context of diabetes, the inflammatory milieu triggers hyperactivation of the JAK–STAT pathway, which specifically manifests as persistent STAT1 phosphorylation. This aberrant STAT1 activity drives the sustained release of pro-inflammatory cytokines (IL-6 and TNF-α) while inhibiting antioxidant responses. Furthermore, the anti-inflammatory arm of this pathway is crippled: the activity of STAT6—a pivotal transcription factor for M2 polarization and tissue repair—is insufficient, thereby impeding the necessary polarization of macrophages towards the reparative phenotype [30, 79–82]. STAT3, a wound healing regulator and immune cell activator, is suppressed in DFUs, which leads to reduced neutrophil/macrophage recruitment and poor control of inflammation [83].

Such dysregulated signalling is further compounded by the suppression of the PPARγ pathway, creating a specific molecular blockade. PPARγ normally acts as a key transcriptional regulator of M2 polarization and exerts anti-inflammatory effects by suppressing cytokines such as IL-6 [84]. However, in diabetic wounds, persistent overexpression of IL-1β decreases PPARγ expression, potentially hindering the transition of macrophages from a pro-inflammatory to a pro-healing phenotype [85]. Crucially, PPARγ and NF-κB operate in a reciprocal inhibitory loop. In the hyperinflammatory environment of diabetic wounds, the constitutive activation of NF-κB overwhelms this balance. High levels of nuclear NF-κB p65 cause the physical sequestration of the PPARγ protein, preventing it from binding to its target peroxisome proliferator response elements (PPREs) [86]. This molecular blockade indicates that unchecked inflammation (NF-κB) actively silences the very machinery (PPARγ) required to resolve it. The inflammatory microenvironment of diabetic wounds imposes a dual blockade of PPARγ by directly decreasing PPARγ expression and indirectly inhibiting its function via NF-κB-mediated antagonism, ultimately leading to the stagnation of macrophage polarization.

Consequently, a vicious cycle is established. The initial hyperglycaemic and inflammatory environment leads to aberrant intracellular signalling, triggering the hyperactivation of STAT1 and the inhibition of STAT6 and PPARγ. This pathway imbalance ‘locks’ macrophages in a pro-inflammatory M1 state, preventing the M1-to-M2 transition. These ‘trapped’ M1 macrophages then secrete even more TNF-α, IL-6, and IL-1β [5, 17, 34, 52], which further suppress PPARγ and cause the hyperactivation of STAT1, perpetually sustaining chronic inflammation state and resulting in nonhealing wounds.

##### Peripheral neuropathy and aberrant secretion of calcitonin gene-related peptide

Peripheral neuropathy affects >50% of individuals with diabetes and is characterized by peripheral nerve dysfunction and reduced intraepidermal nerve fibre density [17, 87, 88]. Diabetic wounds exhibit significantly decreased expression of the sensory nerve-specific marker transient receptor potential vanilloid 1 (TRPV1), suggesting a deficiency in sensory innervation and representing a direct manifestation of diabetic neuropathy at the wound site. Dynamic monitoring of key neuropeptides (calcitonin gene-related peptide, substance P, and vasoactive intestinal peptide) in wound tissues *via* ELISA revealed dysregulated neuropeptide secretion in diabetic wounds compared with normal wounds [89]. Among these neuropeptides, calcitonin gene-related peptide (CGRP) secretion was found to be most significantly reduced in both diabetic mouse models and skin/wound tissues from patients with clinical type 2 diabetes. This reduction disrupts neuroimmune crosstalk—a process essential for tissue healing [89–92].

In healing diabetic wounds, peripheral neuropathy and the associated abnormal secretion of CGRP disrupt macrophage polarization and homeostasis, thereby impeding the resolution of inflammation. Specifically, CGRP deficiency delays the polarization of wound macrophages towards the anti-inflammatory/reparative (M2) phenotype, as indicated by the upregulation of pro-inflammatory cytokines (e.g. IL-1β, TNF-α, IL-6, and IL-12) and the significant downregulation of anti-inflammatory cytokines (IL-10 and TGF-β) and the proangiogenic factor VEGFA. Exogenous CGRP treatment markedly increases the expression of the M2 marker CD206 in macrophages, promotes the secretion of reparative factors such as IL-10, TGF-β, and VEGFA, and suppresses the production of pro-inflammatory cytokines, including TNF-α, IL-6, and IL-1β, thereby directly driving macrophages towards a reparative phenotype [89, 92]. At the molecular level, upon binding to its receptor, CGRP activates adenylate cyclase (AC) via the Gαs protein, leading to a rapid increase in the level of intracellular cyclic AMP (cAMP). cAMP activates protein kinase A (PKA), which phosphorylates the transcription factor cAMP response element-binding protein (CREB) at Ser133. Phosphorylated CREB (p-CREB) is translocated into the nucleus and binds to the CRE in the promoter regions of target genes (e.g. Il10 and Arg1), initiating their transcription [93, 94]. Furthermore, the cAMP–PKA signalling cascade can inhibit the NF-κB-mediated transcription of pro-inflammatory genes through the activation of class IIa histone deacetylases (HDACs). Additionally, the CREB pathway induces the expression of Krüppel-like Factor 4 (KLF4), a key coordinator of M2 polarization that directly activates the M2 gene program while antagonizing the activity of M1-related transcription factors [95]. Thus, deficient CGRP secretion in diabetic wounds specifically suppresses the cAMP–PKA–CREB/KLF4 signalling-dependent M2 polarization program in macrophages, resulting in impaired resolution of inflammation and ultimately delayed tissue repair.

### Consequences of macrophage dysregulation in healing diabetic wounds

Aberrant macrophage polarization impairs normal cellular functions, leading to disrupted signalling between macrophages and epithelial cells, endothelial cells, fibroblasts, and stem cells or tissue progenitor cells. This dysregulation may trigger the disorganization of tissue repair processes, preventing progression to the normal phases of regeneration and ultimately resulting in chronic nonhealing lesions [96, 97].

#### Delayed re-epithelialization

Re-epithelialization deficiency is a hallmark of impaired diabetic wound healing and significantly increases the risks of infection and chronic ulceration. Animal models have demonstrated that wounds in nondiabetic mice achieve near-complete closure by Day 10 and full re-epithelialization by Day 20 postinjury. In contrast, diabetic mice exhibit significantly delayed healing, reaching only 50% closure on Day 10 without the achievement of complete re-epithelialization by Day 20 [34]. Persistent M1 macrophage infiltration and delayed M2 phenotypic transition impair re-epithelialization by suppressing keratinocyte migration and proliferation. Experimental evidence has confirmed that compared with conditioned medium (CM) from M0 macrophages, CM from M1 macrophages markedly inhibits keratinocyte migration, resulting in a 34% reduction in the wound closure rate. Keratinocyte migration is regulated by matrix metalloproteinases (MMPs) and tissue inhibitors of metalloproteinases (TIMPs). M1 CM downregulates MMP1 expression while upregulating TIMP1 expression, establishing an inhibitory microenvironment. The key effector TNF-α, which is secreted by M1 macrophages, mediates migration suppression by inducing TIMP1 overexpression—an effect reversible by TNF-α-neutralizing antibodies [33]. This study elucidated the molecular mechanism by which the dysregulation of macrophage polarization in diabetic wounds impedes re-epithelialization via the TNF-α/TIMP1 axis, providing a theoretical foundation for interventions targeting M1-derived secretory factors or promoting phenotypic switching.

#### Impaired vascularization

Macrophages and their activation states play critical roles in neovascularization during wound repair. Ablation of macrophages during the early or late phases of wound healing disrupts vascular sprouting and regression [98]. During angiogenesis, M2 macrophages exert significant proangiogenic effects, whereas M1 and unpolarized M0 macrophages exert weaker effects [34, 99, 100]. The differential expression of growth factors among macrophage subsets is a key mechanism underlying their distinct angiogenic capacities. Analysis of the gene expression profiles of angiogenesis-related growth factors revealed that M2 macrophages highly express multiple proangiogenic factors, including vascular endothelial growth factor (VEGF), basic fibroblast growth factor (FGF2), insulin-like growth factor-1 (IGF1), chemokine (CCL2), and placental growth factor (PGF) [99, 101–103]. Recent studies have highlighted the pivotal role of matrix metalloproteinase-9 (MMP-9) and its inhibitor TIMP-1 in linking macrophage polarization states to angiogenic mechanisms [104–106]. During their polarization, M2 macrophages downregulate TIMP-1 expression, enabling their secreted proMMP-9 to function in its active form and promote angiogenesis. In contrast, M1 and M0 macrophages, which express high levels of TIMP-1, show reduced proMMP-9 activity, resulting in limited angiogenic capacity. Silencing TIMP-1 in M0 or M1 macrophages significantly increases their proangiogenic potential, validating the central role of TIMP-1 in regulating MMP-9 functionality [100].

However, the roles of macrophage subtypes in angiogenesis remain debated. Studies have suggested that all three activated phenotypes (M1, M2a, and M2c) contribute to angiogenesis but act through distinct mechanisms and during different phases. M1 macrophages function primarily in the initiation phase by secreting known proangiogenic factors such as VEGF. M2a macrophages participate in later stages via platelet-derived growth factor-BB (PDGF-BB) secretion, potentially mediating vascular maturation and stabilization. *In vitro* experiments demonstrated that M2c macrophages robustly promote endothelial cell sprouting and the secretion of large amounts of MMP-9, suggesting their role in vascular remodelling and structural reconstruction. Thus, coordinated interactions between M1 and M2 macrophages are required for optimal wound vascularization [31, 107, 108].

In the pathological microenvironment of diabetic wounds, an imbalance in macrophage polarization profoundly disrupts angiogenesis. Although M1 macrophages secrete proangiogenic factors such as VEGF, their excessive release of inflammatory cytokines (e.g. TNF-α and IL-1β) exacerbates chronic inflammation and impairs endothelial cell function. Furthermore, an insufficient number of M2 macrophages leads to deficits in factors supporting neovessel stability and maturation, such as PDGF-BB and TGF-β. Consequently, diabetic wounds often exhibit poor quality vasculature with an abnormal distribution or structural immaturity [96, 109], further compromising oxygen and nutrient delivery to injured tissues. This ultimately undermines tissue regeneration and perpetuates chronic ulcer formation and delayed healing. Elucidating these mechanisms highlights the therapeutic potential of targeting macrophage polarization to fundamentally improve vascularization in and the healing outcomes of diabetic wounds.

#### Fibrosis and scar formation

The activation of immune responses following tissue injury is an early yet critical process that determines repair outcomes and dictates whether tissue regeneration or scar formation occurs [110]. Macrophages play multifaceted roles in tissue repair, fibrotic mechanisms, and regeneration [111]. Precise regulation of macrophage phenotypes across different phases of tissue repair is essential: reducing the number of pro-inflammatory macrophages or increasing the number of anti-inflammatory reparative macrophages often accelerates tissue repair [97].

M2 macrophages regulate extracellular matrix (ECM) deposition and remodelling, which contribute significantly to fibrotic processes. In schistosomiasis-induced liver fibrosis, M2 macrophages constitute 20%–30% of hepatic granulomas and have been implicated in modulating pathological fibrosis. Other disease models, such as herpesvirus latency-associated fibrosis and silicosis, also demonstrate strong associations with M2 macrophages [111]. Interleukin-4 receptor alpha (IL-4Rα), a key protein that mediates the cellular responses to IL-4 and IL-13 signalling, coordinates the timely transition of macrophages from a pro-inflammatory to an anti-inflammatory phenotype. IL-4Rα-deficient mice exhibit abnormal collagen fibre formation, characterized by an irregular arrangement, reduced density, and significant variability in diameter, with cross-sectional contours displaying irregular shapes [110]. These findings indicate that IL-4Rα deficiency reduces M2 macrophage polarization, disrupting collagen fibre organization in damaged tissues and leading to aberrant fibrosis. Macrophages play dual roles in fibrosis and participate in its suppression, resolution, and reversal. They phagocytose dead cells and debris, reducing the levels of pro-inflammatory and profibrotic mediators. Additionally, macrophages engulf and digest ECM components, stimulating other inflammatory cells (e.g. myofibroblasts and neutrophils) to produce collagen-degrading matrix metalloproteinases (MMPs). M1 macrophage-derived reactive nitrogen species exhibit antifibrotic effects, whereas M2 macrophage-produced IL-10, RELMα, and ARG1 have been shown to suppress fibrosis [111, 112]. To advance the understanding of fibrosis pathogenesis, identifying specific macrophage subpopulations that promote, inhibit, or reverse fibrosis and elucidating the contributions of unique mediators expressed by each subset are critical.

M2 macrophages, while critical for tissue repair in diabetic wounds, exhibit functional impairments that contribute to pathological ECM deposition, fibrosis, and chronic wound formation [43, 113]. Although they are typically associated with anti-inflammatory and pro-repair functions, M2 macrophages in the context of diabetes display aberrant cytokine profiles, including the excessive secretion of transforming growth factor-β1 (TGF-β1) and platelet-derived growth factor (PDGF), which lead to fibroblast hyperactivation and drive abnormal ECM accumulation [12, 113]. Impaired clearance of apoptotic debris by diabetes-associated M2 macrophages further exacerbates inflammation and fibrotic responses [43]. These abnormalities in the ECM disrupt tissue architecture, impede angiogenesis and re-epithelialization, and correlate with imbalances in the expression levels of matrix metalloproteinases (MMPs) and their inhibitors (TIMPs), collectively promoting the persistence of chronic wounds [12, 113]. Additionally, the suppression of anti-inflammatory mediators (e.g. IL-10 and arginase-1) in diabetes-associated M2 macrophages impairs their tissue-reparative capacity [43]. Targeting M2 macrophage dysfunction and its regulatory mechanisms in the context of diabetic fibrosis may offer therapeutic strategies to improve wound healing outcomes.

### Therapeutic strategies targeting macrophage polarization for diabetic wound healing

Diabetic wound management is a complex process for which comprehensive therapeutic strategies and multimodal approaches are needed [41]. Given the pivotal role of macrophage polarization in diabetic wound healing, research in this field has increasingly focused on modulating macrophage phenotypes [114]. While the dysregulation of macrophage polarization in diabetic wounds has been extensively characterized, translating these theoretical insights into effective clinical therapies remains a significant challenge. By integrating current clinical challenges with recent academic advancements, this review categorizes novel therapeutic strategies on the basis of their underlying therapeutic logic, aiming to address three key unresolved challenges in diabetic wounds—signalling silencing, microenvironmental chaos, and insufficient endogenous bioactivity, with the hope that this classification will provide new perspectives for immunomodulatory approaches for the treatment of diabetic wounds.

It should be noted that while the M1/M2 binary paradigm represents a simplified abstraction of a much more continuous macrophage spectrum *in vivo*, recent therapeutic designs operate predominantly within this framework. Therefore, for consistency with the reviewed literature, this section retains the use of this binary paradigm.

#### Precision molecular reprogramming

The persistent activation of pro-inflammatory pathways (e.g. NF-κB and TLR) locks macrophages in the M1 phenotype, creating a molecular barrier to healing. Systemic administration of inhibitors often leads to off-target immune suppression. Consequently, precision molecular reprogramming aims to interfere with these pathogenic signals locally.

##### Cytokine modulation

The extracellular cytokine milieu dictates the macrophage phenotypic switch. In diabetic wounds, this signalling network is severely skewed and characterized by the sustained overproduction of pro-inflammatory cytokines (e.g. TNF-α and IL-6) and a critical deficit of reparative mediators (e.g. TGF-β and IL-10) [23, 27]. Therefore, targeted cytokine modulation serves as a primary strategy to manually rewrite these extracellular cues, correcting the local imbalance to guide macrophages towards the reparative phase.

###### Amplifying M2-polarizing signals

To forcibly initiate the resolution phase, therapeutic strategies have focused on amplifying the signalling of M2-polarizing cytokines, particularly IL-4. IL-4 drives the phenotypic switch via the STAT6 pathway, upregulating the expression of markers such as Arg1 [115]. While direct localized delivery of IL-4 or IL-10 has been shown to increase collagen deposition [116–118], a recent analysis suggested that the efficacy of such approaches depends on the metabolic and receptor sensitivity of target cells. For instance, the glucose transporter GLUT3 has been identified as a critical metabolic checkpoint; its upregulation facilitates the endocytosis of IL-4 receptors, thereby amplifying the IL-4/STAT6 signal [113, 119]. Similarly, the binding of IL-4 to triggering receptor expressed on myeloid cells 2 (Trem2) acts as a ‘brake’ on the pro-inflammatory MAPK/AP-1 pathway [120]. These findings [38, 121] highlight that effective molecular reprogramming requires not only the delivery of cytokines but also the modulation of downstream sensitivity markers (such as GLUT3 and Trem2) to overcome the ‘M2 resistance’ often observed in diabetic tissues.

###### Suppressing pro-inflammatory drivers

Conversely, interrupting the feedback loop of chronic inflammation is necessary for healing. The overexpression of TNF-α is a primary driver of M1 persistence, which directly impairs keratinocyte and endothelial function, leading to severe healing delays [46]. Targeted inhibition of TNF-α, particularly via topical administration, has proven effective in reducing M1 infiltration in perilesional skin [33, 122]. However, simply blocking one cytokine is often insufficient because of pathway redundancy.

###### Dual directional orchestration

As the complexity of the wound milieu has been recognized, advanced strategies now employ a dual direction approach to regulation by simultaneously suppressing pro-inflammatory signals while supplementing reparative factors. For example, the KELE@Gel system demonstrated that natural extracts can broadly suppress a spectrum of cytokines (IL-1β, IL-6, and TNF-α), effectively shifting the macrophage population towards the M2 phenotype [123]. More sophisticated ‘molecular balancing’ is achieved by multifunctional platforms. The MnO_2_ nanozyme-based hydrogel not only scavenges ROS but also mechanistically reprograms the cytokine profile, downregulating CXCL-1 and TNF-α while upregulating IL-4 [124]. Similarly, agents such as calycosin-7-glucoside (CG) [125] and PUHA hybrid scaffolds [126] have been shown to increase the IL-10/TNF-α ratio. This ratio serves as a critical biomarker of the restoration of immune equilibrium, confirming that the most effective molecular strategies are those that orchestrate a comprehensive reset of the inflammatory microenvironment rather than targeting isolated factors.

###### Importance of temporal balance

Favourable wound healing depends on the dynamic balance of macrophage polarization rather than a mere surplus of M2 macrophages, which has been shown to paradoxically delay repair in murine models [127]. Consequently, molecular reprogramming must target the temporal dysregulation of the M1-to-M2 switch to mimic the physiological sequence of macrophage transition in healthy tissues.

##### Pathway interference

While cytokines serve as extracellular messengers, adoption of the distinct phenotype of macrophages is ultimately executed by intracellular signalling networks. Targeting these signalling hubs offers a strategy to ‘short circuit’ the inflammatory program and reprogram cellular metabolism towards regeneration. To provide a structured overview of these mechanisms, the critical signalling pathways, such as NF-κB, JAK–STAT, and HIF, which were selected for their pivotal roles in driving the M1-to-M2 transition, are systematically summarized in Table 3. This table details how specific therapeutic interventions target these molecular checkpoints to reverse the inflammatory blockade characteristic of diabetes and details the resulting biological effects on wound healing (Table 3) [56, 117, 128–133].

**Table 3:** Multiple therapeutic modalities regulate macrophage polarization through targeted modulation of signalling pathways

Signalling pathway	Intervention factor or active ingredient	Target	Upregulate or downregulate	Effects	Ref.
TLR/NF-κB	miR-146a	TRAF6 and IRAK1	Downregulates	Anti-inflammatory	[128]
TLR/NF-κB	Bioactive peptide Andersonin-W1	TLR4, P65 and IκB	Downregulates	Anti-inflammatory, promote re-epithelialization and angiogenesis	[129]
NF-κB	Baicalein (Bai) and berberine (Ber)	IκBα	Downregulates	Anti-inflammatory, promote cell proliferation and angiogenesis, antioxidant	[130]
NF-κB	Platelet-rich plasma (PRP)	IκBα and P65	Downregulates	Anti-inflammatory, promote angiogenesis	[131]
JAK–STAT	Chlorogenic acid and EGF	STAT1, STAT3, and STAT6	Downregulates (STAT1), upregulates (STAT3 and STAT6)	Anti-inflammatory, promote cell proliferation and angiogenesis, antioxidant	[56]
JAK–STAT	IL-10, VEGF, platelet-derived growth factor (PDGF)	STAT3	Upregulates	Anti-inflammatory	[117]
HIF	Platelet-rich plasma (PRP)	HIF-1α	Upregulates	Anti-inflammatory, promote angiogenesis	[131]
TLR/MAPK	miR-632	P38, ERK, and JNK	Upregulates	Anti-inflammatory, promote re-epithelialization	[132]
cGAS–STING	Protein kinase inhibition Y-27632	mtDNA	Downregulates	Anti-inflammatory, promote angiogenesis	[133]

*cGAS–STING* cyclic GMP-AMP synthase–stimulator of interferon genes, *EGF* epidermal growth factor, *ERK* extracellular signal-regulated kinase, *HIF-1α* hypoxia-inducible factor 1-alpha, *IL-10* interleukin-10, *IκB/IκBα* inhibitor of kappa B/alpha, *mtDNA* mitochondrial DNA, *STAT* signal transducer and activator of transcription, *TLR* Toll-like receptor, *TRAF6* TNF receptor-associated factor 6, *VEGF* vascular endothelial growth factor

###### Targeting the TLR/NF-κB axis

The Toll-like receptor (TLR) and NF-κB pathways constitute the primary engines of M1 polarization, a process exacerbated by hyperglycaemia in diabetic wounds [54, 56, 57, 59, 134, 135]. Recent therapeutic interventions focus on severing this link at multiple checkpoints. At the epigenetic level, miR-146a functions as a critical ‘molecular brake’ by targeting TRAF6 and IRAK1; its supplementation effectively dampens downstream NF-κB activity, reversing the deficit observed in diabetic ulcers [128]. Direct biochemical inhibition is also achievable through the use of bioactive agents such as *Astragalus* polysaccharides [130] and PRP-derived factors [131], which block the phosphorylation of IκBα and P65. Crucially, recent findings highlight the importance of temporal precision for pathway modulation. The peptide Andersonin-W1 has a sophisticated dual-phase mechanism: transient activation of TLR4/NF-κB signalling early (Day 3) to aid in debridement, followed by significant suppression of this pathway (Day 8), thereby aligning therapeutic intervention with the physiological phases of repair [129].

###### Rebalancing the JAK–STAT family

Macrophage plasticity is balanced by the JAK–STAT family: STAT1 drives the M1 phenotype, while STAT3 and STAT6 orchestrate M2 polarization [30, 58, 59]. Effective reprogramming requires not inhibiting just one phenotype but also shifting the ratio between them. Innovative responsive materials, such as the OHA-PP@ZCA@EGF hydrogel, achieve this by differentially modulating the chemical microenvironment to suppress STAT1 phosphorylation while simultaneously increasing STAT3 and STAT6 activity [56]. Similarly, the controlled release of IL-10 has been shown to specifically activate the JAK1/STAT3 axis, downregulate inflammation-related genes and solidify the reparative phenotype [117].

###### Modulating broad-spectrum stress sensors

Beyond immune-specific pathways, broad-spectrum stress sensors also dictate macrophage fate. Hyperglycaemia-induced ROS trigger mitochondrial DNA (mtDNA) leakage, which activates the cGAS–STING pathway—a potent driver of sterile inflammation [136]. Interventions using CeO_2_-based nanozymes [133] effectively scavenge excess ROS, preventing mtDNA leakage and thereby blocking the cGAS–STING cascade at its source. Stabilizing HIF-1α expression, for instance, via PRP-based hydrogels, converts hypoxic wound stress into a proangiogenic signal, correcting aberrant polarization while promoting endothelial tube formation [63, 131]. The MAPK pathway acts as a fork in the pathway, where p38 activation favours M1 macrophages and ERK favours M2 macrophages. Peptides such as OA-RD17 exploit this phenomenon by preferentially directing TLR4-mediated signalling towards the ERK pathway, promoting healing without compromising immune surveillance [132].

However, the manipulation of central signalling hubs is associated with the risk of broad-spectrum immunosuppression. Pathways such as NF-κB and TLR are not merely drivers of inflammation but are essential for pathogen recognition and clearance. Indiscriminate inhibition of these axes can inadvertently disarm the innate immune system, leading to unchecked bacterial proliferation in already compromised diabetic tissues [137, 138]. Thus, the therapeutic goal must be the modulation rather than the ablation of these signals, necessitating precise temporal control, which is often lacking with current inhibitors. The future of pathway modulation lies in cell-specific targeting, such as the use of ligand-modified nanocarriers that deliver inhibitors exclusively to macrophages and spare other cell types (e.g. keratinocytes and fibroblasts) to preserve their physiological functions. Moreover, addressing ‘metabolic–epigenetic locking’ requires the development of epigenetic editors (e.g. CRISPR/Cas9-based methylation modifiers) that can permanently erase hyperglycaemic memory from the macrophage genome.

##### Gene regulation

Beyond surface receptors and protein signalling, the most fundamental level of reprogramming occurs at the genetic level, specifically through posttranscriptional regulation via microRNAs (miRNAs). These small noncoding RNAs act as ‘master switches’ capable of silencing entire networks of pro-inflammatory genes simultaneously, offering a distinct advantage over single-target protein inhibitors.

###### Restoring the regulatory landscape

In diabetic wounds, macrophage phenotype dysregulation is often driven by a specific deficit in reparative miRNA signatures. A molecular analysis revealed that restoring these missing genetic brakes is essential for overcoming the M1 lock. For instance, miR-222-3p, which is abundantly expressed in healthy tissues but significantly depleted under diabetic conditions, serves as a critical regulator of immune metabolism; its artificial supplementation directly suppresses M1 markers and restores insulin sensitivity in the local microenvironment [139].

###### Targeting specific signalling axes

The precision of gene therapy lies in its ability to intercept specific inflammatory cascades. For example, miR-4645-5p functions by directly binding to the 3′-UTR of target mRNAs. Mechanistic studies have confirmed that this miRNA effectively silences the MAPKAPK2/TNF-α/SREBP2 axis [140]. The degradation of the mRNA of these upstream drivers prevents the translation of pro-inflammatory proteins before they are synthesized, thereby forcing the cell to default to a reparative M2 state. Recent strategies have thus focused on the design of miRNA mimics and antagomirs [141–143] to act as direct genetic modulators, rewriting the transcriptional program of the macrophage from the inside out.

###### Delivery and off-target challenges

Nevertheless, the application of gene therapy approaches faces substantial delivery hurdles. Naked miRNAs are highly susceptible to nuclease degradation in the wound exudate, and their negative charge hinders cellular uptake. While viral vectors offer efficiency, they raise safety concerns regarding immunogenicity and genomic integration. Moreover, the ‘one-to-many’ nature of miRNA targeting—where a single miRNA modulates hundreds of transcripts—poses a challenge in predicting off-target effects, potentially disturbing unrelated homeostatic networks within the regenerating tissue.

#### Microenvironment normalization

A critical clinical hurdle in diabetic wound management is the hostile microenvironment, characterized by excessive levels of ROS, acidosis, and hyperglycaemia. This toxic milieu not only damages tissue but also degrades therapeutic agents. To overcome this barrier, microenvironment-responsive systems have been developed. These ‘smart’ biomaterials function as both scavengers to neutralize toxicity and dynamic carriers that release drugs only in response to pathological cues (e.g. high ROS levels or low pH).

##### ROS-scavenging strategies

To overcome the limitations of passive antioxidants, the use of nanozymes has emerged as a robust strategy for sustained detoxification. CeO_2_–Y@ZIF-8 nanoparticles [133] exemplify this approach by mimicking the activities of superoxide dismutase (SOD) and catalase (CAT). By catalytically converting toxic superoxide radicals into water and oxygen, these nanoparticles effectively disrupt the oxidative barrier, facilitating a shift towards the M2 phenotype (CD206^+^) and promoting angiogenesis. Crucially, effective scavenging requires precision rather than total elimination, as basal levels of ROS are essential for signalling. Ultrathin WOx nanobelts [144] address this limitation by reducing intracellular ROS levels strictly to reach physiological ranges. This precise modulation prevents metabolic imbalance and restores oxidative phosphorylation (OXPHOS) and ATP production, fundamentally correcting the metabolic defects that drive M1 macrophage persistence.

Beyond chemical catalysis, physical modalities offer external control over the microenvironment. GPBO nanoparticles [145] introduce ‘photothermal–catalytic’ synergy; under NIR irradiation, they not only scavenge ROS but also utilize mild heat to optimize the local redox state, exhibiting excellent biocompatibility and stability across multiple treatment cycles. For a more biological approach, natural product-based strategies such as KELE@Gel [123] leverage the intrinsic antioxidant properties of *Kunzea ericoides* leaf extract. When integrated into a hydrogel matrix, these bioactive components suppress pro-inflammatory cytokines (IL-1β, IL-6, TNF-α) and neutralize free radicals in the environment, providing a gentle yet effective means to restore a pro-reparative immune equilibrium.

A theoretical paradox exists for ROS-scavenging strategies: the ‘oxidative paradox’ is that at physiological levels, ROS are vital signalling messengers for angiogenesis and antimicrobial defence [146, 147]. Quantitatively, eukaryotic cells must maintain an intracellular concentration of H_2_O_2_ within a narrow physiological range of 1–100 nM to execute thiol-based signalling for cell proliferation and migration, which is supported by a steep 100- to 500-fold transmembrane gradient against extracellular levels (1–5 μM). Excessive scavenging (‘over-antioxidation’) that involves decreasing concentrations below these nanomolar thresholds or disrupts the gradient could remove necessary signalling cues from the wound [148, 149]. Future material design must move from ‘total elimination’ to ‘homeostatic modulation’, with the goal of maintaining ROS levels within a range that promotes regeneration rather than depleting ROS entirely. Additionally, the long-term biosafety of metal-based nanozymes (e.g. cerium and manganese) remains a concern, as the potential for heavy metal accumulation and nonbiodegradability may trigger secondary foreign body reactions [150, 151].

##### Smart responsive systems

Static drug delivery systems often fail to adapt to the fluctuating severity of inflammation. Smart responsive systems address this by exploiting the specific pathological cues of the diabetic microenvironment, namely, local acidosis and high ROS levels, as ‘biological keys’ to induce therapeutic release, ensuring temporal and spatial precision. A model of this strategy is the pH/ROS dual-responsive hydrogel (OHA-PP@ZCA@EGF) [56]. This intelligent scaffold, constructed from oxidized hyaluronic acid (OHA) and phenylboronic acid-grafted ε-polylysine (PP), was engineered to remain stable under physiological conditions but undergoes rapid structural disassembly upon encountering the acidic and oxidative milieu of a diabetic ulcer. It utilizes the ‘toxic soil’ itself to trigger the release of epidermal growth factor (EGF) and chlorogenic acid-loaded ZIF-8, ensuring that the therapeutics are concentrated precisely where the inflammatory barrier is most robust. By synchronizing therapeutic action with environmental severity, such systems effectively convert hostile wound signals into a self-regulating repair mechanism, accelerating wound contraction and collagen deposition.

While they are conceptually elegant, the manufacturing complexity and cost of these intelligent biomaterials present barriers to mass production. Furthermore, the ‘trigger thresholds’ for pH or ROS responsiveness are difficult to standardize clinically [152, 153]. Diabetic wounds exhibit significant interpatient heterogeneity; a scaffold tuned to respond to the highly acidic environment of a severe ulcer may fail to release its payload in a less advanced wound, rendering the ‘smart’ system functionally inert in a subset of patients.

#### Supplementation with exogenous bioactive components

In chronic diabetic wounds, endogenous cells are often senescent or functionally impaired and the extracellular matrix is disorganized. Simply reducing inflammation is insufficient; it is essential to replenish the bioactive pool and reconstruct the physical niche required for tissue growth by utilizing stem cells, exosomes, and biomimetic scaffolds.

##### Exosome-mediated bioactive delivery

Extracellular vesicles (EVs), particularly exosomes, have emerged as superior alternatives to live cell therapy. As ‘cell-free’ bioactive packages, they circumvent the risk of immune rejection and limitation of low viability associated with stem cell transplantation in hyperglycaemic environments while retaining the potent paracrine capabilities needed for repair.

Diabetic tissues suffer from signalling silencing. Keratinocyte-derived exosomes (Exosκ-GFP) [154] function as critical messengers that are selectively taken up by macrophages at the wound edge. By delivering a complex payload of proresolving signals, they actively reprogram macrophages from the pro-inflammatory (M1) to the reparative (M2) phenotype, thereby initiating the stalled healing cascade. Similarly, ADSC-derived EVs [155], when delivered via a microneedle patch composed of decellularized adipose matrix, effectively penetrate the tissue barrier. They not only suppress inflammatory cytokines (IL-6 and TNF-α) but also, more importantly, restore the secretion of regenerative factors (Arg1 and IL-10), creating a proangiogenic microenvironment essential for tissue reconstruction.

While natural exosomes show promise, their clinical translation is often hindered by low yield and heterogeneity. To address this, artificial synthetic nanovesicles (Fe-MSC-NVs) [156] represent an important advance in ‘regenerative engineering’. Produced by the extrusion of iron nanoparticle-loaded MSCs through porous membranes, these synthetic vesicles offer high yield and scalability. Crucially, they are engineered to carry more anti-inflammatory cytokines (IL-10) and pro-repair factors (TGF-β3). This ‘enhanced cargo’ strategy directly accelerates wound closure, collagen deposition, and angiogenesis, proving that engineered vesicles can outperform their natural counterparts in restoring the structural integrity of the wound bed.

The transition to exosome-based therapy represents a shift from ‘replacing cells’ to ‘replacing signals’. By delivering the instructions for regeneration (via growth factors and cytokines) rather than the cells themselves, these strategies effectively normalize the senescent microenvironment, enabling the host’s remaining tissue to recover its regenerative potential.

##### Stem cell therapy

While exosomes provide ‘molecular signals’ for repair, stem cell therapy addresses the fundamental deficit of viable, functional cells in the diabetic wound bed. Mesenchymal stem cells (MSCs) [157] are the cornerstone of this approach because of their accessibility and low immunogenicity. Their primary therapeutic efficacy stems from a dual mechanism: direct cell–cell contact and the paracrine secretion of cytokines, which collectively suppress M1 activity and drive M2 polarization [158]. Enhancing the physiological state of hADSCs [159] has been shown to amplify the sustained secretion of critical regenerative factors such as IL-10, TGF-β, and VEGF, thereby driving angiogenesis and collagen synthesis more effectively than unconditioned cells do.

To overcome the metabolic limitations of standard cells, genetic modification has emerged as a powerful strategy to ‘supercharge’ cellular function. A prime example is HPGDS-modified MSCs [160], which are genetically engineered to overexpress haematopoietic prostaglandin D synthase. These enhanced cells actively produce more prostaglandin D2 (PGD2), a metabolite that potently stimulates M2 polarization and accelerates vascularization. These findings demonstrate that the internal genetic machinery of stem cells can generate a more robust therapeutic response than wild-type cells can, specifically in terms of countering diabetes-related impairment.

In addition to traditional MSCs, alternative cell types offer unique regenerative advantages. Human amniotic epithelial cells (hAECs) [161] are notable for their low immunogenicity and high capacity to secrete angiogenic factors, directly increasing capillary density. Furthermore, biological reconstruction strategies employing keratinocyte–fibroblast cocultures [162] focus on restoring the functional skin barrier. By mimicking the natural bilayer structure of skin, these cellular constructs accelerate re-epithelialization and dermal maturation, providing a functional biological cover that purely structural materials cannot replicate.

In addition to traditional MSCs, alternative cell types offer unique regenerative advantages. hAECs [161] are notable for their low immunogenicity and high capacity to secrete angiogenic factors, directly promoting capillary density. Furthermore, biological reconstruction strategies employing keratinocyte–fibroblast cocultures [162] focus on restoring the functional skin barrier. By mimicking the natural bilayer structure of skin, these cellular constructs accelerate re-epithelialization and dermal maturation, providing a functional biological cover that purely structural materials cannot replicate. However, the harsh diabetic microenvironment often leads to poor cell survival and retention [163–165], necessitating advanced delivery strategies to expose the full regenerative potential of these cells. Future strategies must focus on ‘preconditioned’ stem cells (e.g. via hypoxia or pharmacological agents) to increase their stress resistance before transplantation or utilize ‘functionalized organoids’ that provide a protected microniche for transplanted cells [166, 167].

##### Scaffolds and structural support

In the diabetic wound bed, the degradation of the native ECM creates a ‘hostile void’ that prevents cell adhesion, migration, and survival. Therefore, the structural integrity of the tissue must be restored for regeneration. Advanced scaffolds and hydrogels act not only as passive coverings but also as biophysical instructors, exploiting their topography and architecture to direct cellular behaviour and reconstruct the microenvironment.

Microporous scaffolds (MPSs) [159] provide a critical 3D physical niche that mimics the porosity of native tissue. When loaded with hADSCs (hADSC@MPSs), the specific topography solves the problem of ‘cellular homelessness’, significantly improving the retention rate of stem cells in the wound. By providing stable anchorage, this structural support enables these cells to maintain high viability and sustain the secretion of regenerative factors such as VEGF and IL-10, transforming a transient injection of cells into a stable regenerative organoid. Beyond supporting transplanted cells, smart structural matrices can actively initiate the host’s own regenerative potential. The ATAN-Met hydrogel [168] exemplifies this approach via its dynamic network formed by borate ester bonds. This structural design not only fills the defect but also creates a chemically active web (via phenolic hydroxyl groups) that physically captures and recruits endogenous MSCs from the surrounding tissue. Here, the material structure acts as a ‘cellular magnet’, effectively restoring the local cellular population without the need for exogenous transplantation. Restoration also implies the recreation of the stratified complexity of natural skin. Strategies such as bilayer cell sheets [162] move beyond homogenous scaffolds to replicate the specific anatomy of the skin (e.g. keratinocyte–fibroblast layers). This preformed biological architecture provides an immediate structural barrier and signalling gradient, accelerating re-epithelialization more effectively than unstructured therapies do.

The integration of structural support into regenerative restoration marked a crucial evolution from ‘treating the wound’ to ‘rebuilding the tissue’. Physical topography [159] and dynamic bonds [168] function as essential permissive factors that allow biological therapies (cells and exosomes) to exert their effects. In the absence of such structural restoration, biological signalling is often lost in the chaotic environment of diabetic wounds, resulting in the establishment of a cohesive, pro-regenerative microenvironment.

However, the introduction of synthetic scaffolds carries the inherent risk of eliciting a foreign body response. If the degradation rate of the scaffold does not match the rate of tissue regeneration or if the material stiffness does not match that of the native skin, the implant may be enclosed by a fibrotic capsule rather than integrated. This encapsulation prevents immune cell infiltration and vascularization [169, 170], effectively turning the therapeutic scaffold into a physical barrier that hinders, rather than helps, wound closure.

#### Translational bottlenecks and future strategic perspectives

Despite the significant potential demonstrated by macrophage-targeted immunomodulatory strategies in preclinical models, none have successfully transitioned into routine clinical practice. To bridge this translational gap, fundamental challenges in terms of scientific conceptualization, therapeutic design, validation methodologies, and practical implementation need to be overcome.

A major scientific limitation is the persistence of the outdated M1/M2 binary paradigm. The diverse therapeutic strategies reviewed here undeniably demonstrate the translational utility of targeting macrophage behaviour. However, their reliance on the rigid M1/M2 binary paradigm underscores a major conceptual limitation, ultimately leading to certain failures in clinical translation. Single-cell RNA sequencing (scRNA-seq) has revealed a pathological continuum of macrophage activation states in diabetic wounds characterized by ‘transcriptional stall’ or mixed phenotypes [127]. Consequently, future precision therapies must move beyond inducing broad M2 polarization. Instead, they must precisely identify and target these dysfunctional intermediate subsets to resolve epigenetic blockade and restore cellular plasticity, effectively shifting the goal from flipping a switch to tuning a spectrum.

The complexity of diabetic wounds demands parallel evolution in therapeutic design. The pathological microenvironment is highly heterogeneous and shaped by variable degrees of ischaemia, neuropathy, and infection [171], rendering ‘one-size-fits-all’ immunomodulatory approaches inadequate. The next goal lies in the development of intelligent theranostic platforms that integrate real-time sensing of microenvironmental cues (e.g. pH, proteases, and ROS) for feedback-controlled, adaptive drug delivery [172–174]. This dynamic ‘sense-and-respond’ capability is crucial for administering immunomodulators within the optimal therapeutic window, paving the way for truly personalized wound management.

Progress is further constrained by the limitations of existing preclinical models. The disconnect between rodent healing, which occurs primarily by contraction, and human healing, which occurs via re-epithelialization, coupled with the failure of acute models to replicate chronic human wound features such as cellular senescence and complex biofilms [175–178], contributes to high clinical attrition. Increasing translational predictability requires a shift towards high-fidelity systems. Porcine models, humanized skin organoids, and *in vitro* microphysiological systems offer more relevant platforms for accurately simulating human macrophage pharmacokinetics and rigorously evaluating the biological efficacy of immunomodulatory strategies [179, 180].

Finally, the durability and safety of these advanced therapies present formidable challenges for implementation. The hostile diabetic wound milieu poses a dual challenge: the induction of macrophages with a reparative phenotype are prone to reversion under sustained metabolic stress [181, 182], and sophisticated biomaterials face a ‘protease storm’ of MMPs and ROS that can lead to unpredictable degradation kinetics [183, 184]. Material stability must be carefully engineered; degradation that is too rapid risks toxic burst release, while degradation that is too slow impedes tissue integration. Furthermore, mismatches in the degradation rate or mechanical properties can trigger a foreign body response, leading to fibrotic encapsulation that ultimately defeats the therapeutic purpose. Overcoming these barriers is essential for translating promising mechanisms into lasting clinical solutions.

## Conclusions

In summary, chronic nonhealing diabetic ulcers represent a systemic failure of immune orchestration wherein macrophages are pathologically locked in a pro-inflammatory M1 state by the complex interplay of hyperglycaemic memory, oxidative stress, and impaired hypoxic signalling. To overcome this therapeutic bottleneck, this field is undergoing a profound paradigm shift towards precision immunomodulation, utilizing smart microenvironment-responsive biomaterials, stem cells, and engineered exosomes to neutralize local toxicity and replenish the regenerative signalling niche. Moving forward, successful clinical translation demands moving beyond the oversimplified binary M1/M2 model to precisely target the continuous macrophage activation spectrum revealed by single-cell multiomics. Ultimately, by engineering dynamic, adaptive delivery systems capable of real-time sensing, next-generation therapies can achieve the spatiotemporal precision necessary to recreate the natural sequence of healing and liberate the body’s intrinsic regenerative capacity, transforming wound stagnation into true tissue regeneration.
